# Prevalence of Ischemic Heart Disease Among Urban Population of Siliguri, West Bengal

**DOI:** 10.4103/0970-0218.44518

**Published:** 2009-01

**Authors:** Sukanta Mandal, Joyti Bikash Saha, Sankar Chandra Mandal, Rudra Nath Bhattacharya, Manashi Chakraborty, Partha Pratim Pal

**Affiliations:** Department of Community Medicine, North Bengal Medical College, Sushrutanagar, Darjeeling, West Bengal, India; 1 Department of Cardiology, North Bengal Medical College, Sushrutanagar, Darjeeling, West Bengal, India

**Keywords:** IHD, risk factor, urban population

## Abstract

**Objectives:**

To determine the prevalence of ischemic heart disease and the associated risk factors among the urban population of Siliguri.

**Materials and Methods:**

A cross-sectional survey of a random sample of the population aged ≥40 years old in the Municipal Corporation area of Siliguri. Study variables were age, sex, occupation, addiction, food habit, physical activity, body mass index, blood pressure, and electrocardiogram change.

**Results:**

Out of 250 individuals who took part in this study, 29 (11.6%) had ischemic heart disease (IHD) and 118 (47.2%) had hypertension. Males had a higher (13.5%) prevalence of IHD than females (9.4%). About 5% of the patients had asymptomatic IHD. IHD among the study population is significantly associated with hypertension and smoking.

## Introduction

Cardiovascular disease, one of the non-communicable diseases, has become a major public health problem in many developing countries. About two-thirds of the global estimated 14.3 million annual cardiovascular disease deaths occur in the developing world. By the year 2015, cardiovascular diseases could be the most important cause of mortality in India. The prevalence of coronary artery disease in India increased from 1% in 1960 to 9.7% in 1995 in urban populations, and in rural populations it has almost doubled in the last decade.(2) There is an epidemiological transition from infective to degenerative diseases, increases in the prevalence of cardiovascular risk factors, and ageing of the population, which eventually leads to an increase in the absolute numbers of people with coronary heart disease (CHD) and increased health awareness and demand for health care facilities. However, reports on the prevalence or incidence of ischemic heart disease (IHD) in developing countries including India are very scarce, and routinely collected data are often incomplete and unreliable.(1,2,3,9) This study was done to determine the prevalence of IHD and associated risk factors among the urban population.

## Materials and Methods

A cross-sectional survey of a random sample of the population aged ≥ 40 years old in the Municipal Corporation area (adult population = 472374) of Siliguri was conducted.(13) The sample size of 246 was calculated by using Epi-Info software, Version 3.3.2 assuming an expected prevalence of IHD among the urban population in India is 9.7%, the worst acceptable prevalence is 6%, confidence level 95%. Considering a non-response of 10%, a sample size of 271 individuals was decided.

There are 47 wards in the Siliguri Municipal Corporation area. Ward number 23 (Dabgram) and 47 (Pati colony) were selected from the 47 wards of Siliguri. The total population of two selected wards was 14,568. From the updated voter list of this ward, all the members who were permanent residents aged ≥ 40 years old were selected. By using the systematic random sampling method, a list of 271 members aged ≥ 40 years old was prepared with names and addresses. They were given a pre-tested, semi-structured questionnaire (the Rose questionnaire from the cardiovascular survey methods of WHO-1982).(14) Necessary data was collected after obtaining informed consent. The smoking habit was stratified according to the number of cigarettes smoked per day and the duration of smoking. Dietary data was collected through interviews regarding type of food (vegetarian or non-vegetarian).

For physical activity, the definition of the Indian consensus group was used according to which a person is considered to have a sedentary behavior if he walks less than 14.5 km a week.(14) Body mass index (BMI) was computed as weight in kg/meter^2^. Obesity was defined as a BMI of >27 kg/m^2^ and overweight was defined as a BMI of >25 kg/m^2^. Figures for criteria laid down by the Indian consensus group for being overweight (>23.5 kg/m^2^) were also calculated. Hypertension was diagnosed when the systolic blood pressure was 140 mmHg or more and the diastolic blood pressure was 90 mmHg or more, as per the guidelines of the British Hypertension Society.(19) Twelve lead electrocardiograms (ECGs) were taken using a BPL 108 ECG machine on each individual. Each ECG was reviewed by a cardiologist. A maximum of three visits were conducted for those individuals who could not be contacted during the first visit.

## Criteria for the Diagnosis of Ischemic Heart Disease

The criteria for the diagnosis of ischemic heart disease were: (a) a history of angina or infarction and previously diagnosed disease, (b) an affirmative response to the Rose questionnaire, and (c) electrocardiographic findings, namely Minnesota codes 1-1, 4-1, 5-9, 5-2 or 9-2.(14) The presence of all three criteria were taken as confirmation of the diagnosis of coronary artery disease. The prevalence of coronary artery disease was also classified according to the presence or absence of symptoms. Those who knew they had the disease or showed an affirmative response to the Rose questionnaire were classified as symptomatic patients.

## Data Analysis

Quantitative data was entered into EPIINFO software, Version 3.2 and then exported to SPSS software, Version 10.0 for analysis. Data was analyzed and tabulated using frequency distribution tables and proportion. Association between the prevalence of IHD and risk factors were examined using the Chi-square test. EPIINFO software, Version 3.2 was used to calculate Chi-square for the linear trend of change in IHD. Binary logistic regression analysis was done between the predictor variable and dichotomous outcomes (ischemic heart disease or not).

## Results

Of the 271 individuals age ≥40 years old enrolled in the study, 250 took part in the study. The mean age of the study population was 52.8 years old (+ 12.6). The mean age of males was 54 years old and the mean age of females was 51.5 years old. Among 250 study subjects, 29 (11.6%) had IHD. The prevalence of IHD significantly increased with an increase in age (*P*<0.01). The highest prevalence of IHD was found in the ≥ 80 years old group (40.0%) and the lowest prevalence was found in the 40–49 year old group (5.4%). The prevalence of symptomatic IHD was 6.4% and that of asymptomatic IHD was 5.2% [Table 1]. Prevalence of IHD was higher among males (13.5%) as compared with females (9.4%)(*P*>0.05).

**Table 1:** The prevalence of ischemic heart disease in different age groups among the study population

Age groups (years)	Study population	Symptomatic (Known + Rose questionnaire) (A)	Electrocardiographic (Silent CAD) (B)	Total (A+B)	Odds ratio
40–49	128 (51.2)	4 (3.1)	3 (2.3)	7 (5.4)	1
50–59	46 (18.4)	3 (6.5)	2 (4.3)	5 (10.8)	2.11
60–69	40 (16.0)	3 (7.5)	2 (5)	5 (12.5)	2.47
70–79	26 (10.4)	4 (15.35)	4 (15.35)	8 (30.7)	7.68
≥80	10 (4.0)	2 (20)	2 (20)	4 (40.0)	1152
Total	250	16 (6.4)	13 (5.2)	29 (11.6)	

Chi-square for linear trend = 19.13; *P* value < 0.01; Figures in parentheses are in percentages

The overall prevalence of hypertension among the study population was 47.2%. Among the patients with hypertension, the prevalence of Grade 2 hypertension (18.4%) was higher than Grade 1 (14.4%). Grade 3 hypertension was 6%, Grade 1 isolated systolic hypertension was 5.6%, and Grade 2 isolated systolic hypertension was 2.8% [Table 2].

**Table 2:** Distribution of study population according to their blood pressure

	Blood pressure	Frequency	Percentage (%)
Non-hypertensive	Normal	92 (36.8)	-
	High-normal	40 (16.0)	52.8
Hypertensive	Grade 1 hypertension	36 (14.4)	47.2
	Grade 2 hypertension	46 (18.4)	-
	Grade 3 hypertension	15 (6.0)	-
	Isolated systolic hypertension (Grade 1)	14 (5.6)	-
	Isolated systolic hypertension (Grade 2)	7 (2.8)	-
	Total	250	100.0

The prevalence of IHD among smokers was higher than among non-smokers (*P*<0.01) [Table 3]. Prevalence of IHD increases with the increase in blood pressure (*P*<0.01). The highest prevalence of IHD was found among the severe hypertensive population (26.7%) and the lowest prevalence was found in those patients with normal blood pressure (3.3%). The prevalence of IHD increased with higher BMI (*P*<0.05). The prevalence was 25% among patients with BMI ≥ 30 and 6.8% among patients with BMI 18.5-23.5.

**Table 3:** Distribution of the study population according to coronary risk factors and the presence of ischemic heart disease (n = 250)

Coronary risk factors	Ischemic heart disease		Total	OR	*P* value
				
	Yes	No			
Smoking habit						
Yes	18 (20.45)	70 (79.55)	88 (100)	3.53	
No	11 (6.70)	151 (99.30)	162 (100)	1	0.0012
Hypertension					
Normal	3 (3.3)	89 (96.7)	92 (100)	1	
High normal	3 (7.5)	37 (95.0)	40 (100)	2.41	
Grade 1 hypertension	7 (19.4)	29 (80.6)	36 (100)	7.16
Grade 2 hypertension	10 (21.8)	36 (78.2)	46 (100)	8.24	
Grade 3 hypertension	4 (26.7)	11 (73.3)	15 (100)	10.79	
Isolated systolic hypertension (Grade 1)	1 (7.1)	13 (92.9)	14 (100)	2.28	
Isolated systolic hypertension (Grade 2)	1 (14.3)	6 (85.7)	7 (100)	4.94	0.00008
BMI (kg/m^2^)					
< 18.5	4 (9.1)	40 (90.9)	44 (100)	1	
18.5-23.5	7 (6.8)	95 (93.2)	102 (100)	0.74	
23.5-25	5 (11.9)	37 (88.1)	42 (100)	1.35	
25-30	8 (19.05)	34 (80.95)	42 (100)	2.35	
≥ 30	5 (25.0)	15 (70.0)	20 (100)	3.33	0.03

Figures in parentheses are in percentages. OR = Odds Ratio

Binary logistic regression analysis shows that 88% of IHD can be explained by predictor variables. Diastolic blood pressure, systolic blood pressure, and smoking habits were significantly associated with IHD, whereas no significant relation was found between age, sex, physical activity, body mass index, or diet habit [Table 4].

**Table 4:** Association between ischemic heart disease and coronary risk factors by binary logistic regression analysis

Predictor variable	Standard error	Wald	*P* value	Expected
Age	0.021	0.776	0.378	1.019
Sex	0.585	0.194	0.660	0.773
Body mass index	0.032	0.526	0.468	1.023
Diastolic blood pressure	0.025	8.724	0.003	1.077
Systolic blood pressure	0.011	5.093	0.024	1.026
Smoking habit	0.621	4.995	0.025	0.250
Diet	0.957	0.279	0.597	1.658
Physical activity	0.227	0.618	0.713	1.255
Constant	2.793	25.555	0.000	0.000

## Discussion

This study shows that the prevalence of coronary artery disease was 11.6% in the urban populations, which is lower than the Trivandrum study (13.9%) and the Tirupati study (12.63%). Lower prevalence was reported in Chennai (11%).(15,18) Sex-specific prevalence of CAD in the present study was more or less similar to the Trivandrum study. However, higher prevalence of CAD was observed in females in the Tirupati study (6.86% vs. 15.81%).(5,15) The prevalence of CHD reported in different studies [Table 5] showed that the prevalence of coronary artery disease has almost doubled in the rural areas and increased nine-fold in the urban populations, and that the rates are higher in South India compared to the north.(5–9)

**Table 5:** A comparative analysis between this study and other studies with regard to the reported prevalence of coronary heart disease

Author	Study year(s)	Region	Residence	Age group (years)	Sample size	Prevalence rate (%)
Present study	2005	Siliguri, West Bengal	Urban	≥ 40	250	11.6
Latheef, *et al*.(17)	2006	Tirupati	Urban	≥ 20	1519	12.63
Gupta, *et al*.^(22)^	2002	Jaipur	Urban	≥ 20	1123	8.2
Mohan, *et al*.(18)	2001	Chennai	Urban	≥ 20	1262	11
ICMR study(2)	1989-94	Delhi	Urban and rural		Urban-3019	Urban-7.6
					Rural-2434	Rural-5.3
ICMR study(2)	1989-94	Vellore	Urban and rural	25–64	Urban-2649	Urban-4.0
					Rural-4693	Rural-1.5
Beegom, *et al*.(5)	1995	Trivandram	Urban	25–65	506	13.9
Gupta, *et al*.(2)	1994	Rajasthan	Rural	25–64	1905	4.6
		Trivandrum,	Urban	-	-	Urban-13.9
Kutty, *et al*.(4)	1993	Kerala	Rural	25–64	1130	Rural-4.6
Chaddha, *et al*.(16)	1990	Delhi	Urban	25–64	13275	9.7

In the Jaipur study, a 3.2% prevalence of symptomatic CAD (known coronary artery disease plus Rose-positive angina) was similar to the Delhi survey (3.2%) but higher prevalence (6.4%) was noted in the present study.(3) The higher prevalence (5.2%) of asymptomatic/unreported infarction and angina pectoris in both sexes in our study appears to be due to illiteracy and ignorance about heart attacks in Indians. Economic status may also not allow them to approach a cardiologist for diagnosis and treatment. In developed countries, no rural-urban differences exist in the prevalence of coronary artery disease and coronary risk factors. The prevalence of coronary artery disease was approximately 10% between 25 and 64 years of age in most of the developed countries, which may be slightly higher in the United States and Northern Europe and lower in Southern Europe, Japan, and Australia.(10) The Seven Countries Study showed that the force of a risk factor may vary from one population to another, which may be explained by the presence of possible protective factors, such as physical activity, and adverse factors such as dietary saturated fat and antioxidant deficiency. There was a higher prevalence of risk factors in the urban population as compared with rural subjects, but the prevalence is less than in developed countries, although the prevalence of coronary artery disease is comparable.(21) However, sample size was not sufficient for comparative analysis of risk factors. Some other coronary risk factors like diabetes and lipid profile were also not included due to resource constraints.

## Conclusions

The findings of this study indicate that the prevalence of coronary artery disease and coronary risk factors is high in this urban population in India. Further research is required to document the impact of increased physical activity, change in dietary habit to more quantities of fruits and vegetables and reduced use of poly unsaturated fatty acid, no smoking, and controlling blood pressure and cholesterol as is recommended in the third joint task force guidelines of European and other societies on cardiovascular disease prevention in clinical practice.(21)
